# Gibberellin-Stimulation of Rhizome Elongation and Differential GA-Responsive Proteomic Changes in Two Grass Species

**DOI:** 10.3389/fpls.2016.00905

**Published:** 2016-06-23

**Authors:** Xiqing Ma, Bingru Huang

**Affiliations:** ^1^College of Agro-grassland Science, Nanjing Agricultural University, NanjingChina; ^2^Department of Plant Biology and Pathology, State University of New Jersey, New Brunswick, NJUSA

**Keywords:** gibberellic acid, hydroponics, Kentucky bluegrass, plant proteomics, tall fescue

## Abstract

Rapid and extensive rhizome development is a desirable trait for perennial grass growth and adaptation to environmental stresses. The objective of this study was to determine proteomic changes and associated metabolic pathways of gibberellin (GA) -regulation of rhizome elongation in two perennial grass species differing in rhizome development. Plants of a short-rhizome bunch-type tall fescue (TF; *Festuca arundinacea*; ‘BR’) and an extensive rhizomatous Kentucky bluegrass (KB; *Poa pratensis*; ‘Baron’) were treated with 10 μM GA_3_ in hydroponic culture in growth chambers. The average rhizome length in KB was significantly longer than that in TF regardless of GA_3_ treatment, and increased significantly with GA_3_ treatment, to a greater extent than that in TF. Comparative proteomic analysis using two-dimensional electrophoresis and mass spectrometry was performed to further investigate proteins and associated metabolic pathways imparting increased rhizome elongation by GA. A total of 37 and 38 differentially expressed proteins in response to GA_3_ treatment were identified in TF and KB plants, respectively, which were mainly involved in photosynthesis, energy and amino acid metabolism, protein synthesis, defense and cell development processes. Accelerated rhizome elongation in KB by GA could be mainly associated with the increased abundance of proteins involved in energy metabolism (glyceraldehyde-3-phosphate dehydrogenase, fructose-bisphosphate aldolase, and ATP synthase), amino acid metabolism (*S*-adenosylmethionine and adenosylhomocysteinase), protein synthesis (HSP90, elongation factor Tu and eukaryotic translation initiation factor 5A), cell-wall development (cell dividion cycle protein, alpha tubulin-2A and actin), and signal transduction (calreticulin). These proteins could be used as candidate proteins for further analysis of molecular mechanisms controlling rhizome growth.

## Introduction

Rhizomes are underground stems with meristematic tissues in rhizome nodes capable of generating shoots and roots, which also serve as an important storage organ for carbohydrates, nutrients and water in perennial grass species (Jernstedt and Bouton, 1985; Li and Beuselinck, 1996). Recent studies have shown that some genes and proteins are highly enriched or specifically expressed in rhizome tissues, including energy and metabolism related genes, such as monosaccharide transporter and methionine *S*-methyltransferase in wild sorghum (*Sorghum propinquum*; Jang et al., 2006), glucose-6-phosphate/phosphate translocator, fructose-bisphosphate aldolase, sucrose synthase, and oligosaccharyl transferase in wild rice (*Oryza longistaminata*; Hu et al., 2011; He et al., 2014; Zhang et al., 2014), and ATP synthase subunit and UDP-glycosyltransferase in lotus (*Nelumbo nucifera*) rhizome (Cheng et al., 2013). Defense-related genes such as peroxidase, L-ascorbate peroxidase, glutathione *S*-transferase and catalase have also been identified in wild rice rhizomes and lotus rhizomes (Cheng et al., 2013; Zhang et al., 2014). Some growth-related regulators are found to be enriched in rhizomes, such as the elongation factor, tubulin alpha-3 chain, growth regulating factor in wild rice (He et al., 2014; Zhang et al., 2014), nuclear RNA-binding proteins, mitogen-activated protein kinase, proteasome regulatory particle triple-A ATPase subunit and elongation factor in wild sorghum (Jang et al., 2006), and cell division control 20 and histone H4 in sacred lotus (Kim et al., 2013). These studies suggest that aforementioned proteins and genes could play important roles in rhizome development.

Rapid and extensive rhizomes development is a desirable attribute for rapid canopy establishment and persistence or tolerance to biotic and abiotic stresses (Tao et al., 2001; Sacks et al., 2006; Su et al., 2008; Zhou et al., 2014). Some perennial grass species, such as Kentucky bluegrass (KB; *Poa pratensis*), develop extensive rhizomes and are referred to as rhizomatous grasses while other grass species propagate mainly through tillering with no or small rhizomes, such as tall fescue (TF; *Festuca arundinacea*), which are known as bunch-type grasses (Turgeon, 2011). Rhizomatous grasses can persist through prolonged periods of drought and exhibit superior post-drought recovery than tilling- or bunch-type grasses (Su et al., 2008; Turgeon, 2011; Zhou et al., 2014). However, the underlying mechanisms or metabolic factors regulating rhizome growth in perennial grass species are largely unknown.

Among phytohormones, GA plays central roles in controlling cell elongation and stem elongation (Hedden and Kamiya, 1997). For example, with exogenous GA_3_ treatment, the length of rice seedlings showed significant increases compared with that of the untreated control; further studies indicated that the abundance of glyceraldehyde-3-phosphate dehydrogenase (GAPDH), alpha ATPase subunit, catalase, calreticulin and cell division cycle protein 48 (CDC48) were increased with GA_3_ treatment (Wang et al., 2013). For rhizome development, several GA-related genes exhibit greater abundance in rhizomes compared to regular stems, such as GA 20 oxidase and GA-regulated protein in lotus rhizomes (Cheng et al., 2013), GA signal transduction protein (SPINDLY) in bamboo (*Phyllostachys praecox*; Wang et al., 2009), and GA 2-beta-dioxygenase, GA regulated protein, and GA receptor GID1 in wild rice rhizomes (Hu et al., 2011; He et al., 2014). These results suggested that rhizome development may be mediated by GA. As discussed above, perennial grass species, such as TF and KB exhibit distinct features of rhizome development; however, whether differential rhizome growth in grass species is mediated by differential responses of rhizomes to GA or the mechanisms of how GA may regulate rhizome growth in perennial grass species are not well-understood.

We hypothesize that rhizomatous KB is more responsive to GA than short-rhizome TF, which could be due to differential expression of proteins involved in metabolic processes controlling cell growth. The objective of this study was to investigate whether rhizomes of TF and KB respond differently to GA and determine major metabolic processes which could be associated with the differential responses through proteomic analysis.

## Materials and Methods

### Plant Materials and Growth Conditions

Tall fescue (cultivar ‘BR’) and KB (cultivar ‘Baron’) plants were collected from the turfgrass research farm at Adelphia, NJ and transplanted to plastic trays (54 cm × 27 cm × 6 cm) filled with fritted clay in a greenhouse. During the 2 months establishment phase, plants were irrigated three times per week, and fertilized weekly with half-strength Hoagland’s nutrient solution (Hoagland and Arnon, 1950). Plants were kept at 6–7 cm canopy height by mowing weekly. The greenhouse had an average temperature of 20°C and 780 μmol m^-2^ s^-1^ of photosyntheitcally active radiation (PAR) with natural sunlight and supplemented with sodium lump on cloudy days.

After establishment, TF and KB plants with the same number of tillers without rhizomes and roots were transferred to the hydroponic system within plastic boxes (56 cm × 54 cm × 15 cm), each containing 20 L half-strength Hoagland’s solution in a controlled growth chamber (Conviron, Winnipeg, MB, Canada). Each box had 40 individual plants wrapped in sponge strips and held in position with foam board and aerated with an electric pump (Tetra, Blacksburg, VA, USA). Nutrient solution was changed every 4 days and pH was monitored every day and maintained at 6.5. The growth conditions consisted of a 14 h-photoperiod, temperature of 20/18°C (day/night), and PAR of 680 μmol m^-2^ s^-1^ at the canopy level.

### Treatments and Experiments Design

After plants acclimated to the hydroponic condition for 5 days, hormone treatment was imposed. Thirty plants in each container were maintained in either half-strength Hoagland’s solution alone (untreated control) or with 10 μM GA_3_ dissolved in the nutrient solution (GA_3_ treatment); this rate was based on previous experimental results (Ma et al., submitted). The untreated control and GA_3_ treatment were replicated in four containers (four replicates per treatment), which were arranged in a randomized complete block design.

Prior to hormone treatment (0 day), and at 6 and 12 days of treatment, the length of rhizomes of each plant was measured. Rhizome length was measured on 30 individual plants from each replicate in each treatment.

### Protein Extraction and Two-Dimensional Electrophoresis Separation

The rhizome tissues, collected from each treatment at 12 days of GA_3_ treatment and washed three times with deionized water, were immediately frozen in liquid nitrogen and stored at -80°C for proteomic analysis. Protein extraction was performed according to Xu et al. (2010), Kamal and Komatsu (2016a) with modification. About 1.5 g tissues were homogenized in 6 ml ice-cold precipitation solution (10% TCA, 0.07% 2-mercaptoethanol in acetone), then precipitated overnight at -20°C. Following this, pelleted proteins were centrifuged for 15 min at 4°C and 9, 000 *g*, and washed three times with a rinse solution (0.07% 2-mercaptoethanol in acetone) to remove pigments and lipids. The pellets were then air dried and suspended with resolubilization buffer [8 M urea, 1% CHAPS, 1% IPG buffer (GE Healthcare), 2 M thiourea, 1% dithiothreitol (DTT)], and sonicated for 1 h at 4°C. The concentration of extracted proteins were quantified according to Bradford (1976) with a commercial dye reagent (Bio-Rad Laboratories, Hercules, CA, USA) using a bovine serum albumin protein standard.

First dimension isoelectric focusing was carried out using an IPGphor apparatus (GE Healthcare, Piscataway, NJ, USA). Immobiline DryStrips (pH 3–10, GE Heathcare, Piscataway, NJ, USA) were rehydrated with 250 μg of protein samples (total 250 μl) in a rehydration solution of 8 M urea, 1% CHAPS, 1% IPG buffer (GE Healthcare), 2 M thiourea, 1% dithiothreitol, and 0.002% bromophenol blue. The voltage for rehydration and focusing were set at 50 V for 14 h, 500 V for 1 h, 1000 V for 1 h, 5000 V for 1 h, and 8000 V to a total of 80 kVh. Following isoelectric focusing equilibration was performed using an equilibration buffer of 50 mM Tris-HCl (pH8.8), 6 M urea, 2% sodium dodecyl sulfate (SDS), 0.002% bromophenol blue, 30% glycerol, and 1% DTT for 15 min two times and then incubated for another 20 min using the same solution which contained 2.5% iodoacetamide instead of DTT. For second dimensional electrophoresis a Hoefer SE 600 Ruby electrophoresis unit (GE Healthcare, Piscataway, NJ, USA) was used with a 12.5% SDS-polyacrylamide gel. Electrophoresis was performed at 5 mA per gel for 45 min and then increased to 18 mA per gel for 7 h. Then gels were stained using a Coomassie Brilliant Blue G-250 (CBB) solution as described by Neuhoff et al. (1988), and were scanned with a Typhoon FLA 9500 (GE Healthcare, Piscataway, NJ, USA). Each treatment was replicated four times from four independent tissue extractions. Gel images were analyzed using SameSpots software (v4.5, Nonlinear USA, Inc., Durham, NC, USA). Protein spots were normalized to the total volume of spots on the gel and automatically aligned. Only protein spots with corresponding *p*-values less than 0.05 were selected for further analysis when hormone treatments were compared against the untreated controls.

### Protein Identification and Functional Analysis

The differentially expressed proteins (DEPs) were excised manually and washed with 30% acetonitrile (ACN) in 50 mM ammonium bicarbonate before DTT reduction and iodoacetamide alkylation as according to Xu et al. (2008). An 8 μl of 10 μg ml^-1^ Trypsin (Promega Sequencing Grade, Modified Trypsin) dissolved in 25 mM NH_4_HCO_3_ at pH 8.0 was used to digest proteins in each gel spot for overnight at 37°C. The resultant peptides were extracted with 30 μl of a 1% trifluoracetic acid extraction buffer, concentrated with ZipTip (Millipore, Corp., Bedford, MA, USA), and then mixed in a 1:1 ratio with 7 mg mL^-1^ α-cyano-4-hydroxy-cinnamic acid. The peptides were analyzed on a 4800 MALDI TOF/TOF analyzer (Applied Biosystem, Framingham, MA, USA) that was equipped with a 200 Hz, 355 nm UV laser and calibrated with a mixture of peptides. Mass spectra (m/z 880–3200) were acquired in positive ion reflector mode. Peaks with an S/N > 25 were pick out for subsequent MS/MS sequencing analysis in the 1 kV mode. Proteins were identified using the Peptide Mass Fingerprinting module of Mascot search engine (V2.2, Matrix Science, Boston, MA, USA) against the green plant NCBI database. The parameters were set to: trypsin, methionine oxidation, carboxyamidomethylation of cysteine, two missed cleavages, precursor mass tolerance of 50 ppm, and fragment mass tolerance of 0.6 Da. Confidence interval values of >95% for at least two peptides were considered as being successfully identified.

For identified protein’s classification and functional analysis, protein homologs were identified against the *Arabidopsis* database^1^, and gene ontology (GO) was categorized against the agrigo database^2^ for biological process and cellular component analysis; threshold was set to -log10 > 4. Functional classification of proteins mainly involved in energy, metabolism, stress defense, regulatory, and unknown proteins based on the classifications of Bevan et al. (1998), and protein interactions analysis were performed by STRING 10^3^. The parameters were set to: active interaction sources (textmining, experiments, databases, co-expression, neighborhood, gene fusion, and co-occurrence), minimum required interaction score (medium confidence), meaning of network edges (confidence).

### Gene Expression Analysis of Differentially Expressed Proteins

Total RNA was extracted from the same treatments for protein analysis using Trizol-reagent (Life Technologies, Grand Island, NY, USA) according to the manufacturer instructions, and treated with DNase (TURBO DNA-free kit, Ambion, Austin, TX, USA). A 2 μl total RNA was reverse transcribed using High-Capacity cDNA Reverse Transcription Kit (Life Technologies, Grand Island, NY, USA). Power SYBR Green PCR Master Mix (Life Technologies, Grand Island, NY, USA) was performed for cDNA amplification on the StepOnePlus Real-Time PCR System (Applied Biosystems, Foster City, CA, USA). The EST sequences of KB were kindly provided by Gan et al. (2016), and the details of primer sequences were provided in Supplementary Table S1.

### Statistical Analysis

All data were subjected to analysis of variance according to the general linear model of SAS 9.0 (SAS Institute, Inc., Cary, NC, USA), and two-way ANOVA analysis was used by SPASS 13.0 for Windows (SPSS, Chicago, IL, USA). Treatment means were separated using the Fisher’s protected least significant difference (LSD) test at *p* ≤ 0.05.

## Results

### Genetic Variation and GA_3_ Effects on the Elongation of Rhizomes

**FIGURE 1:**
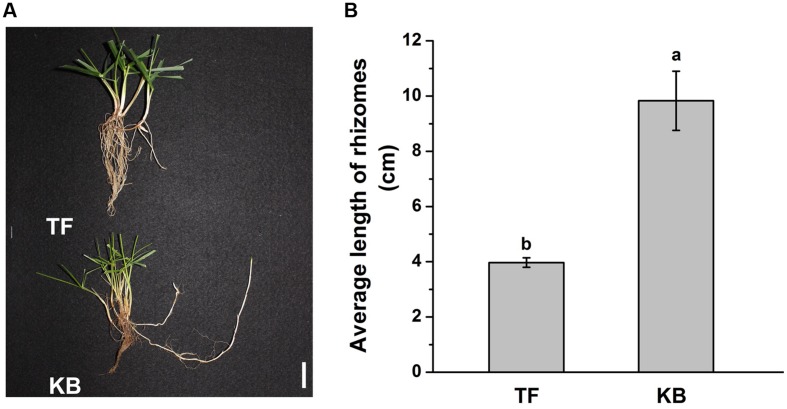
**Rhizomatous Phenotypes of the tall fescue (TF) and Kentucky bluegrass (KB) **(A)**, and average length of rhizomes in greenhouse conditions (B).** The values represent the mean ± SE of 30 rhizomes. Columns marked with different letters indicated significant differences among treatments based on the LSD value (*p* ≤ 0.05). Bar represents 2 cm.

**FIGURE 2:**
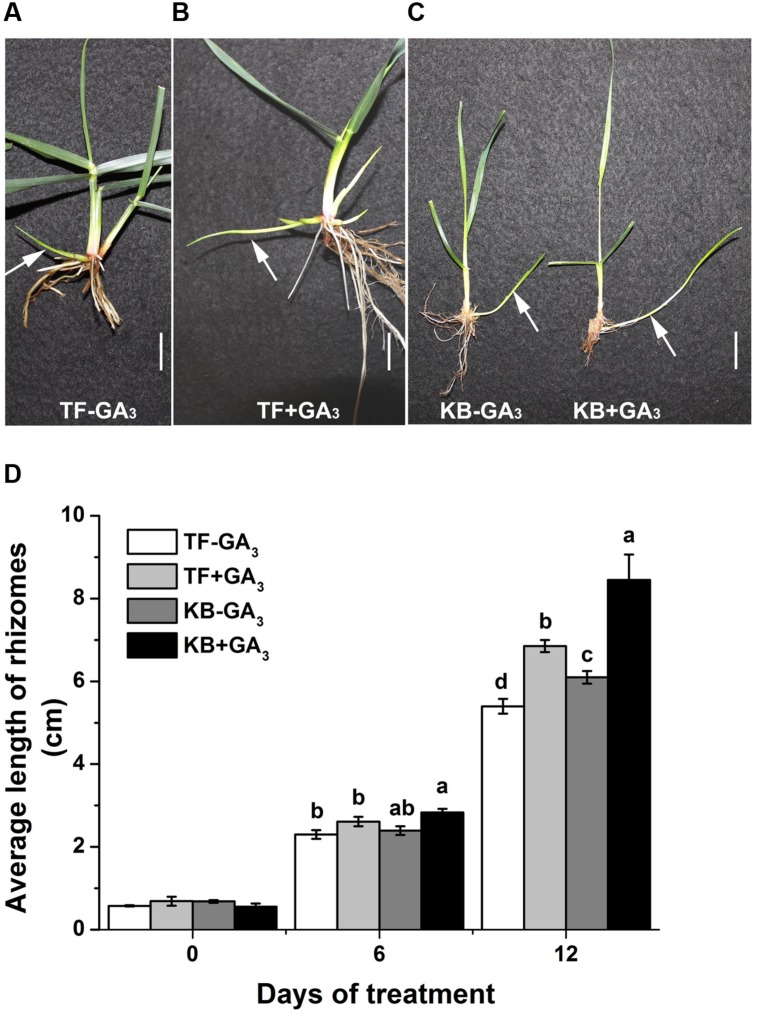
**Effects of GA_3_ on the rhizome growth in perennial grass. (A,B)** Rhizomatous phenotypes of TF with GA_3_ treatment in hydroponics at 12 days in growth chamber, white arrow indicated rhizomes. Bar represents 2 cm. **(C)** Rhizomatous phenotypes of KB with GA_3_ treatment in hydroponics at 12 days in growth chamber, white arrow indicated rhizomes. Bar represents 2 cm. **(D)** Average length of rhizomes in hydroponics. The values represent the mean ± SE of 30 rhizomes. Columns marked with different letters indicated significant differences among treatments at a given day based on the LSD value (*p* ≤ 0.05).

Rhizomatous phenotypes of 3-months-old TF and KB plants are illustrated in **Figure 1A**. The length of rhizomes was 3.97 ± 0.17 and 9.83 ± 1.07 cm for TF and KB plants, respectively, for 3 months-old plants (**Figure 1B**). At 6 days of transplanting to the hydroponic system, there was no significant difference in the length in rhizomes between these two species with or without 10 μM GA_3_ treatment (**Figure 2D**). However, after 12 days of GA_3_ treatment, the length of rhizomes of KB was increased by 38.52% (from 5.40 ± 0.18 to 6.85 ± 0.14 cm) while it increased by 26.85% (from 6.10 ± 0.15 to 8.45 ± 0.61 cm) in TF plants compared with the untreated control, respectively (**Figures 2A–D**).

### Differential Protein Accumulation in Rhizomes between TF and KB in Response to GA_3_ Treatment

**FIGURE 3:**
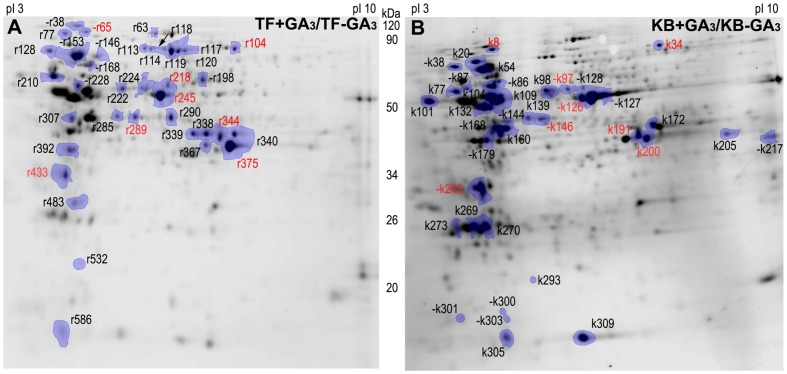
**Two-dimensional SDS-PAGE gels of rhizome with GA_3_ treatment in TF **(A)** and KB (B).** Differentially expressed proteins (DEPs) were marked between treatments, each treatment with at least four repeats gels (*p* ≤ 0.05). “–” indicates protein down-regulated with GA_3_ treatment compared with the untreated control. Red color represents common regulation proteins between TF and KB.

To further investigate proteins and associated metabolic processes that could be related to differential GA-stimulation of rhizome elongation in the two grass species, protein profiles were performed using two-dimensional electrophoresis. 530 proteins were detected in rhizomes of TF. Among them, 37 GA-responsive DEPs were identified, including 30 up-regulated proteins and seven down-regulated proteins with GA_3_ treatment (**Figure 3A**; Supplementary Table S2). 400 proteins were detected in KB rhizomes, and among those, 38 GA-responsive DEPs were identified, including 21 up-regulated and 17 down-regulated proteins with GA_3_ treatment (**Figure 3B**; Supplementary Table S2). There were eight conserved proteins identified in both species with GA_3_ treatment (**Figure 3**).

### Functional Classification and Subcellular Localization of the Identified Proteins

**FIGURE 4:**
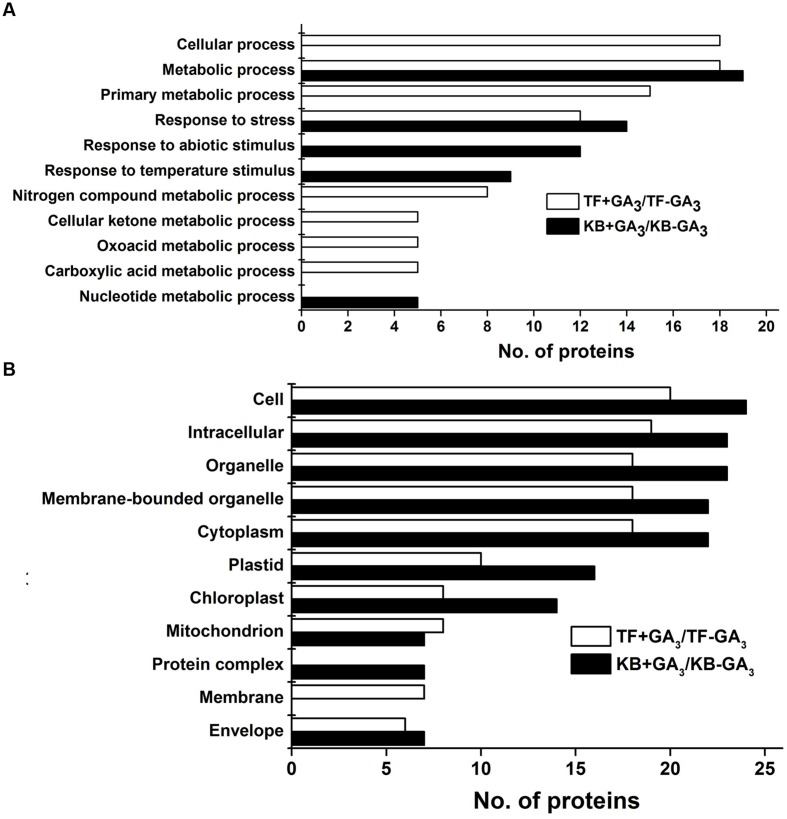
**Cluster analysis of DEPs in rhizomes with GA_3_ treatment in TF and KB.** Biological process **(A)** and cellular component **(B)** were analyzed against the *Arabidopsis* database (http://bioinfo.cau.edu.cn/agriGO/analysis.php; threshold: -log10 ≥ 4).

**FIGURE 5:**
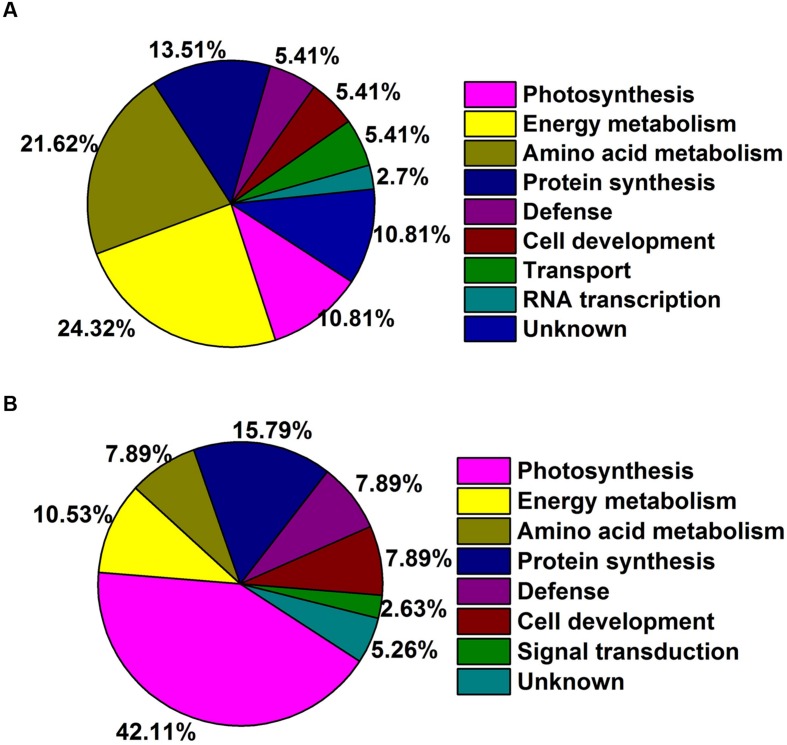
**Functional categories DEPs of rhizomes response to GA_3_ treatment in TF **(A)** and KB **(B)** based on Bevan et al. (1998) categorization.** Percentages based on total identified proteins, including both up and down-regulated proteins.

Gene ontology was performed to analyze the biological processes and cellular process of proteins differentially expressed as regulated by GA in the two species. Abundant proteins found in TF rhizomes included those with functions in response to stress and several metabolic intermediates processes (including those involved with nitrogen compounds, cellular ketones, oxoacid and carboxylic acid). Proteins involved in metabolic process, response to stress, abiotic stimulus and temperature stimulus were found in KB rhizomes with GA_3_ treatment (**Figure 4A**). For cellular component analysis, there was no difference between these two species with GA_3_ treatment in rhizomes tissues; they both contained proteins localized to cell, intracellular spaces, organelles, membrane bounded organelles, cytoplasm, plastid, chloroplast, mitochondrion, membrane and envelopes (**Figure 4B**). Based on the functional analysis, the GA_3_-responsive proteins in TF were mainly categorized into photosynthesis (10.81%), energy metabolism (24.32%), amino acid metabolism (21.62%), protein synthesis (13.51%), defense (5.41%), cell development (5.41%), transport (5.41%), RNA transcription (2.7%), and unknown proteins (10.81%; **Figure 5A**); the GA_3_-responsive proteins in KB were mainly categorized into photosynthesis (42.11%), energy metabolism (10.53%), amino acid metabolism (7.89%), protein synthesis (15.79%), defense (7.89%), cell development (7.89%), signal transduction (2.63%), and unknown proteins (5.26%; **Figure 5B**).

### Proteins Involved in Photosynthesis

**FIGURE 6:**
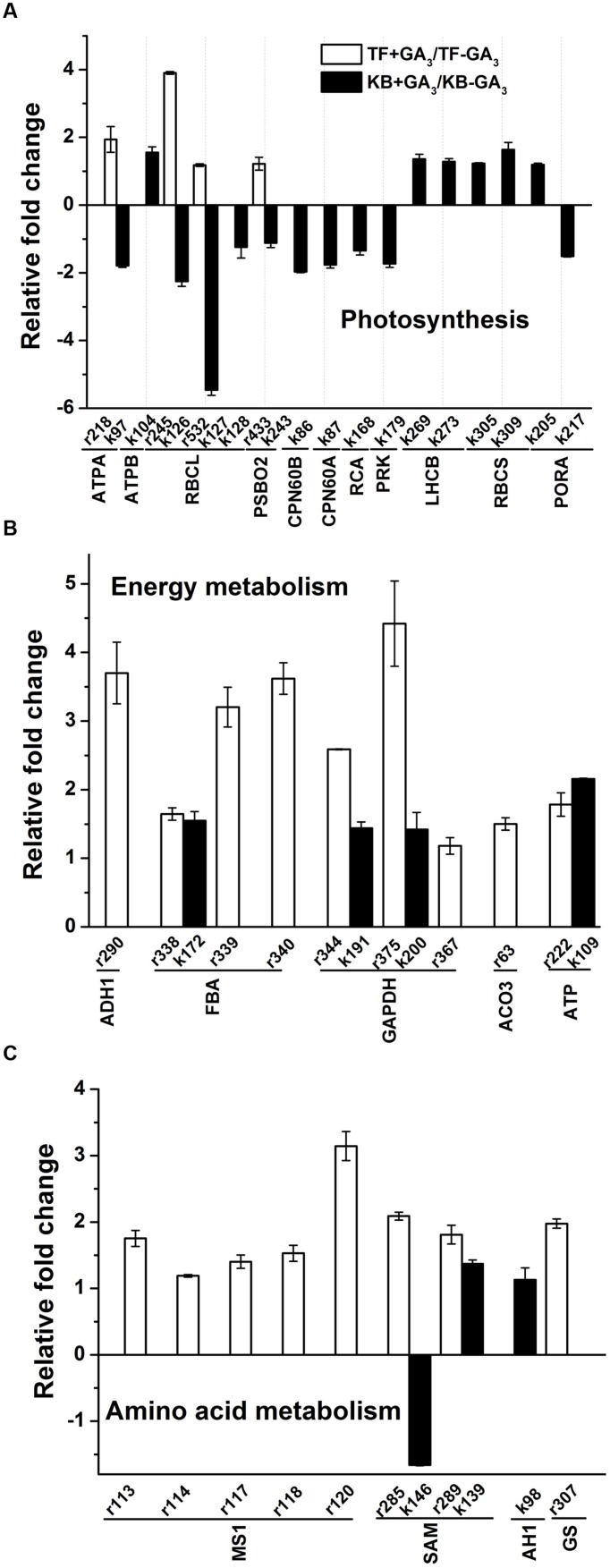
**Up-regulation and down-regulation of protein spots in response to GA_3_ treatment represented as fold change compared to the untreated control.** Charts are organized by the functional category of protein spots, and include Photosynthesis **(A)**, Energy metabolism **(B)**, Amino acid metabolism **(C)**. Spot numbers correspond to the spot numbers in Supplementary Table S2 and **Figure 3** that are the protein spots with significantly altered accumulation according Fischer’s LSD (*p* ≤ 0.05). The values represent mean ± SE (*n* = 4 replicates per treatment).

For photosynthesis-related proteins, the abundance levels of four proteins were increased, including ATP synthase subunit alpha (ATPA, r218) by a 1.94-fold increase, RuBisCO large subunit (RBCL, r245, r532) by 3.90 and 1.18-fold increases, and oxygen-evolving enhancer protein 1 (PSBO2, r433) by a 1.22-fold increase with GA_3_ treatment compared the untreated controls in TF plants (**Figure 6A**). For KB plants treated with GA_3_, 10 out of 16 protein abundant levels were decreased compared to the untreated control, including ATPA (k97) by 1.79-fold, RBCLs (k126, k127, and k128) by 1.24 to 5.46-fold, PSBO2 (k243) by 1.12-fold, RuBisCO large subunit-binding protein (CPN60B, k86) by 1.97-fold, RuBisCO large subunit (CPN60A, k87) by 1.76-fold, RuBisCO activase (RCA, k168) by 1.34-fold, phosphoribulokinase (PRK, k179) by 1.73-fold, NADPH-protochlorophyllide oxidoreductase (PORA, k217) by 1.51-fold. Another six proteins abundance levels were increased, including ATP synthase subunit beta (ATPB, k104) by 1.55-fold, chlorophyll a-b binding proteins (LHCB, k269, k273) by 1.30-fold, ribulose bisphosphate carboxylase small chain (RBCS, k305, k309) by 1.23 to 1.64-fold, PORA (k205) by 1.19-fold (**Figure 6A**).

### Proteins Involved in Energy Metabolism

For energy metabolism, the abundance levels of all differentially accumulated proteins were increased with GA_3_ treatment in TF plants, including alcohol dehydrogenase 1 (ADH1, r290) by 3.70-fold, fructose bisphospohate aldolase (FBA, r338, r339, and r340) by 1.65 to 3.62-fold, GAPDH (r344, r375, r367) by 1.18 to 4.42-fold, ATP synthase subunit alpha (ATP, r222) by 1.79-fold and aconitate hydratase (ACO3, r63) by 1.50-fold when compared with the untreated control (**Figure 6B**). With GA_3_ treatment in KB plants, the abundance levels of four proteins were increased, including FBA (k172) by 1.55-fold, GAPDH (k191, k200) by 1.40-fold, ATP (k109) by 2.16-fold compared with the untreated control, respectively (**Figure 6B**).

### Proteins Involved in Amino Acid Metabolism

For amino acid metabolism, the abundance levels of eight proteins were increased with GA_3_ treatment in TF plants, including methionine synthase 1 (MS1, r113, r114, r117, r118, and r120) by 1.19 to 3.14-fold, *S*-adenosylmethionine synthase (SAM, r285, r289) by 1.81 to 2.09-fold, and glutamine synthetase (GS, r307) by 1.98-fold when compared with the untreated control (**Figure 6C**). With GA3 treatment in KB plants, the abundance level of SAM (k146) was decreased by 1.66-fold, and the abundance levels of two proteins increased, including SAM (k139) by 1.37-fold, and adenosylhomocysteinase (AH1, k98) by 1.13-fold when compared with the untreated control (**Figure 6C**).

### Proteins Involved in Protein Synthesis

**FIGURE 7:**
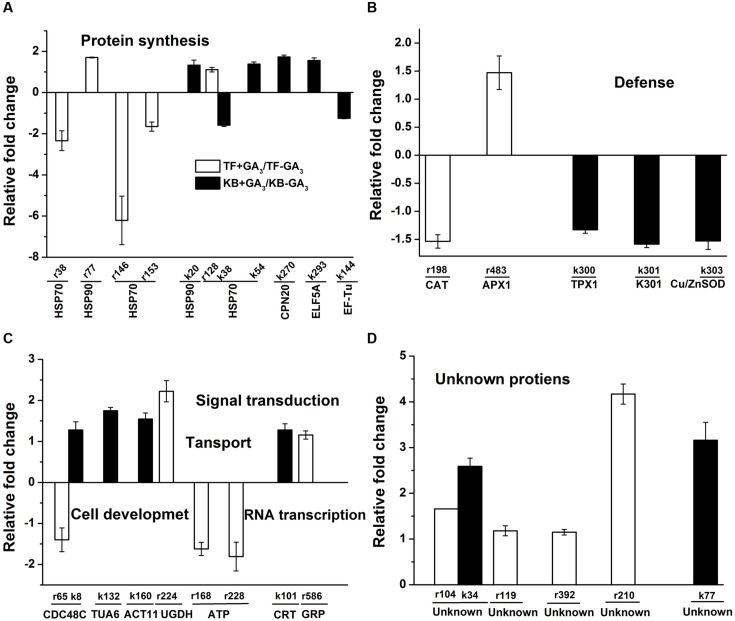
**Up-regulation and down-regulation of protein spots in response to GA_3_ treatment represented as fold change compared to the control treatment.** Charts are organized by the functional category of protein spots, and include protein synthesis **(A)**, defense **(B)**, regulatory related proteins **(C)**, and unknown **(D)** functions. Spot numbers correspond to the spot numbers in Supplementary Table S2 and **Figure 3** that are the protein spots with significantly altered accumulation according Fischer’s LSD (*p* ≤ 0.05). The values represent mean ± SE (*n* = 4 replicates per treatment).

For proteins involved in protein synthesis, the abundance level of 70 kDa heat shock related protein (HSP70, r38, r146, and r153) was decreased by 1.65 to 6.21-fold, while the abundance levels of heat shock 90 kDa protein (HSP90, r77) and HSP70 (r128) were increased by 1.70 and 1.11-fold, respectively, in GA_3_ treatmented TF plants when compared with the untreated control (**Figure 7A**). With GA_3_ treatment in KB plants, the abundance levels of four proteins were increased, including HSP90 (k20), HSP70 (k54), 20 kDa chaperonin (CPN20, k270) and eukaryotic translation initiation factor 5A (ELF5A, k293) by 1.33, 1.38, 1.73, and 1.55-fold, respectively, compared with the untreated control while the abundance levels of HSP70 (k38) and elongation factor Tu (EF-Tu, k144) were decreased by 1.58 and 1.25-fold, respectively, compared with the untreated control (**Figure 7A**).

### Proteins Involved in Defense Related Proteins

For defense related proteins, GA_3_ treatment in TF plants decreased the abundance level of catalase (CAT, r198) by 1.54-fold, while the abundance level of ascorbate peroxidase (APX1, r483) was increased by 1.47-fold compared with the untreated control. With GA_3_ treatment in KB plants, the abundance levels of three defense related protein spots were decreased, including peroxiredoxin 5 cell rescue protein (TPX1, k300), oxidoreductase protein (k301) and superoxide dismutase[Cu-Zn] (k303) by 1.33, 1.59, and 1.53-fold, respectively, compared with the untreated control (**Figure 7B**).

### Proteins Involved in Cell Development

For proteins involved in cell development, GA_3_ treatment in TF plants decreased the abundance level of cell division cycle protein (CDC48C, r65) by 1.41-fold, and increased the abundance level of UDP-glucose 6-dehydrogenase (UGDH, r224) by 2.22-fold compared with the untreated control (**Figure 7C**). With GA_3_ treatment in KB plants, the abundance levels of three protein spots were increased, including CDC48C (k8), alpha tubulin-2A (TUA6, k132) and actin-1 (ACT11, k160) by 1.28, 1.75, and 1.55-fold, respectively, compared with the untreated control (**Figure 7C**). the abundance level of several proteins involved in transport were decreased with GA_3_ treatment, including vacuolar proton-ATPase subunit (ATP, r168) and ATP binding protein (ATP, r228) by 1.62 and 1.81-fold, respectively, compared with the untreated control in TF. The abundance level of calreticulin (CRT, k101) involved in signal transduction in KB samples with GA3 treatment was increased by 1.28-fold compared with the untreated control, and the abundance level of a glycine-rich RNA-binding protein (GRP, r586) involved in RNA transcription was increased by 1.16-fold with GA_3_ treatment in TF samples when compared with the untreated control (**Figure 7C**).

Additionally, six protein spots with unknown functions also had significant increases between the two perennial grass species with application GA_3_ treatment compared with the control; the variations of abundance were increases by 1.18- to 4.17-fold (**Figure 7D**).

### Analysis of the Differentially Expressed Proteins at mRNA Level

**FIGURE 8:**
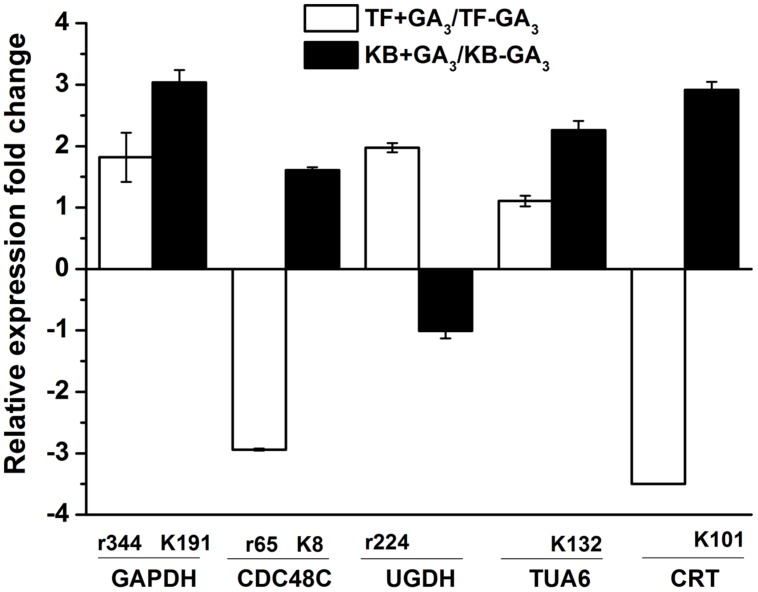
**Gene expression levels of the DEPs.** Charts are organized by relative expression fold change of corresponding protein spots in response to GA_3_ treatment represented as fold change compared to the control treatment. The values represent mean ± SE (*n* = 4 replicates per treatment).

To further confirm the protein expression changes during rhizomes development with GA_3_ treatment between TF and KB, the transcription levels involved in energy metabolism, cell development, and signal transduction proteins were investigated (**Figure 8**). For GAPDH (r344, k191), with GA_3_ treatment, the gene expression levels increased by 1.81 and 3.03-fold in TF and KB compared with untreated control, respectively. CDC48C (k8) expression increased by 1.61-fold in KB with GA_3_ treatment compared with the control, while CDC48C (r65) expression decreased by 2.94-fold in TF with GA_3_ treatment compared with the control, which were in accordance with the corresponding protein pattern. In addition, UGDH (r224) increased 1.97-fold in TF with GA_3_ treatment compared with untreated control, TUA6 (k132) and CRT (k101) increased 2.26 and 2.91-fold in KB with GA_3_ treatment compared with the control, respectively. It should be noted that UGDH (KB), TUA6 (TF), and CRT (TF) expression were up-regulated or down-regulated, although the abundance of corresponding protein spots were not identified with GA3 treatment. The correlation coefficient between these proteins and corresponding genes expression is 0.84.

## Discussion

Mechanisms controlling rhizome formation and growth are not well-understood, particularly for perennial grass species. Our previous study indicated that the transcript levels of ent-kaurene oxidase, GA 20-oxidase1 and DELLA involved in GA metabolism and signaling pathways were increased in rhizomes with GA_3_ treatment compared with the control in TF, expansin and endotransglucosylase/hydrolases family genes responsible for GA treatment were also increased with exogenously applied GA_3_ in TF rhizomes (Ma et al., submitted). In the present study, two-way ANOVA analysis indicated that differences of DEPs response to GA treatment in these two perennial grass species were mainly affected by GA treatment (**Table 1**). Furthermore, comparative proteomic analysis showed that the underlying mechanisms of GA-regulation of rhizome elongation are complex in both rhizomatous KB and short-rhizome TF, involving multiple differentially or commonly regulated metabolic processes, mainly including energy metabolism, amino acid metabolism, photosynthesis, regulatory proteins, and protein–protein interactions (PPIs). Biological functions of GA-responsive proteins associated with the promotion of rhizome elongation are discussed below.

**Table 1:** Two-way ANOVA analysis of species (TF and KB) and GA_3_ treatment interactions.

Source	df	*F*	*p*
Species	1	3.023	0.101
GA_3_	1	9.013	0.008
Species * GA_3_	1	0.655	0.430
Error	16		

In the present study, with exogenous application GA_3_, photosynthesis related proteins such as ATP synthase, RBCL, PSBO2 were identified to be differentially regulated in these two species. Several corresponding proteins were also identified in rhizomes of wild sorghum and wild rice. For example, the enrichment of RBCL was identified in the rhizome tips of *S. propinquum* (Jang et al., 2006); chlorophyll a oxygenase and LHCB were down-regulated in the rhizome tips relative to shoot tips of *O. longistaminata* (Jang et al., 2006; Hu et al., 2011). Furthermore in our study, 10 out of 16 proteins involved in photosynthesis decreased in KB (**Figure 6A**); it has been reported that the promotion of leaf elongation of rice by GA_3_ treatment was associated with the suppression of photosynthesis-related proteins including RBCS (Wang et al., 2013). Since rhizomes are underground stems capable of generating daughter plants for photosynthesis, the suppression of photosynthesis-related genes in rhizomes before daughter plants are formed may be beneficial for rhizome elongation by allocating more energy to growth-related proteins (Jang et al., 2006; Hu et al., 2011; He et al., 2014), thereby, more down-regulation enrichment involved in photosynthesis proteins in KB with GA_3_ treatment maybe positively regulate rhizome elongation compared to that of in TF.

In our study, almost all proteins related to energy metabolism and amino acid metabolism increased in the abundance in rhizomes of TF and KB in response to GA_3_ treatment, including FBA, GAPDH, ACO3, MS1, SAM, AH1, and GS. FBA is a glycolytic enzyme, and a previous study indicated that the activity of FBA was increased in roots of rice seedling by exogenous GA_3_ treatment (Konishi et al., 2004). GAPDH is involved in oxidation and phosphorylation of D-glyceraldehyde-3-phosphate to D-1, 3- biphosphoglycerate, while no research has reported its change with GA treatment in rhizomes, GAPDH was found to be up-regulated in rice during stimulated elongation of leaves treated with GA_3_ treatment (Tanaka et al., 2004; Komatsu et al., 2006). ACO3 catalyzes the inter-conversion of citrate to isocitrate is involved in tricarboxylic acid cycle in the mitochondria (Ding et al., 2012). Furthermore, MS and SAM catalyzing the synthesis of methionine and adenosylmethionine involved in the biosynthesis of many secondary metabolites, such as polyamines which were found to increases in elongating internodes of stolons in strawberry (*Fragaria × ananassa*; Fang et al., 2011; Hildebrandt et al., 2015). GS is a key enzyme involved in ammonium assimilation and catalyzes the synthesis of glutamine from ammonium and glutamate (Lothier et al., 2011). The induction or up-regulation of those proteins involved in energy and amino acid metabolism by GA_3_ treatment could be related to GA-promotion of rhizome elongation in perennial grass.

Heat shock proteins, as co-chaperones, play essential roles involved in protein folding, assembly, translocation, and degradation (Wang et al., 2004). In our study, the abundance of HSP90, HSP70, CPN20, and ELF5A were increased in rhizomes of KB with GA_3_ treatment. EIF5A is mainly involved in RNA metabolism and trafficking, and regulates cell proliferation and cell growth (Thompson et al., 2004). A previous study also demonstrated that a defect of *ELF5A* could cause plant dwarfism and abnormal floral organs in *Arabidopsis* (Feng et al., 2007). CPN20, a chloroplast co-chaperonin, has been shown to mediate iron superoxide dismutase activity in *Arabidopsis* chloroplasts (Kuo et al., 2013). In the present study, increased abundance of these proteins also possibly increased downstream proteins abundance including CDC48C, TUA6, ACT11, and CRT in rhizome of KB plants; it has been shown that the abundance level of CDC48 was increased by application GA_3_ treatment in rice seedlings (Wang et al., 2013). Additionally, actin and tubulin are cytoskeleton components involved in cell division and cell elongation, which has been shown to be up-regulated in association with application of GA_3_ in rice leaf sheath (Yang and Komatsu, 2004; Kamal and Komatsu, 2016b). Calreticulin (CRT), a major Ca^2+^-sequestering protein, also has been proven to regulate rice leaf sheath elongation involved in GA signaling pathways (Shen et al., 2003). The up-regulation of the regulatory proteins involved in chaperons and cell wall development could contribute to the pronounced stimulation of rhizome elongation by GA_3_ treatment in KB.

**FIGURE 9:**
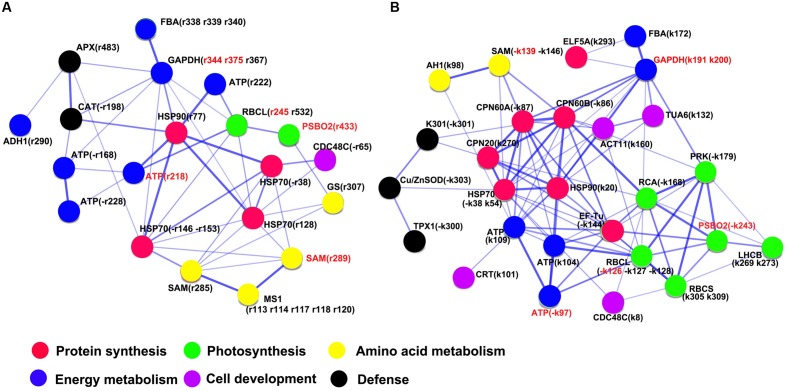
**Network of putative protein–protein interactions with GA_3_ treatment in TF **(A)** and KB **(B)** compared to the control using the STRING 10.0 pathway analyzer.** The circles represent overrepresented proteins. The blue lines represent the putative interaction between proteins. The density of edges represents confidence score of putative functional association. Spot numbers correspond to the spot numbers in Supplementary Table S2 and **Figure 3** that are the protein spots with significantly altered accumulation (*p* < 0.05).

Furthermore, PPIs were analyzed using STRING analyzer dependent on the physical and functional analysis, which showed that HSP90 could be a central protein in both KB and TF plants (**Figures 9A,B**). The structure of HSP90 was composed of N terminal ATP-binding domain, middle domain and C-terminal dimerization domain (Xu et al., 2012). The major role of HSP90 is not only to manage protein folding, but also plays important roles involved in signaling-transduction, cell-cycle control, protein degradation, and trafficking (Wang et al., 2004). In this study, 30 out of 37 DEPs exhibited putatively interactive relationship in the rhizome of TF plants, and the proteins complexes of HSP90, HSP70, and ATPase could positively regulate photosynthesis responsive proteins (ATP, RBCL, and PSBO2), energy metabolism (FBA and GAPDH), amino acid metabolism (MS1, SAM, and GS) and defense-related proteins (CAT and APX1), and negatively regulate CDC48C synthesis (**Figure 9A**). Meanwhile, 34 out of 38 DEPs could putatively interact in the rhizome of KB plants; the proteins complexes including HSP90, HSP70, CPN60, CPN20, and ATPase could positively regulate CDC48C, TUA6, ACT11, and CRT synthesis, and negatively regulate photosynthesis responsive proteins (RCA, PRK, RBCL, RBCS, LHCB, and PSBO2) and defense-related proteins (K301, TPX1, and Cu/ZnSOD; **Figure 9B**). Thereby, positive interactions between chaperones and cell-wall development proteins may possibly promote rapid elongation of rhizomes in KB with GA3 treatment.

**FIGURE 10:**
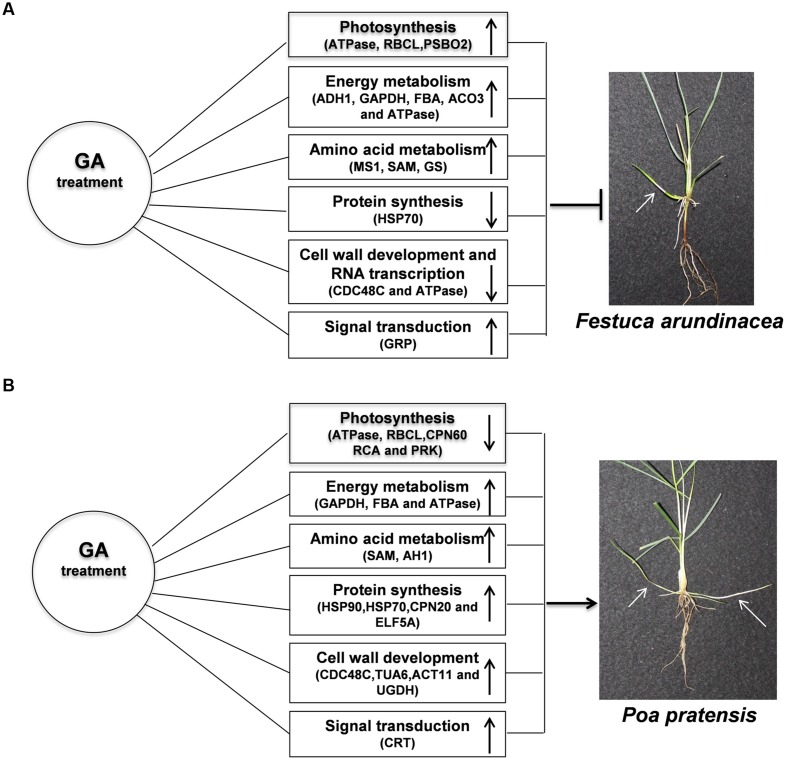
**Model for GA-responsive rhizomes elongation of TF **(A)** and KB (B).** White arrows indicated rhizomes.

Taken together, rhizomes elongation was promoted in both perennial grass species with exogenous application of GA_3_ treatment (**Figures 10A,B**); however, more pronounced responses of leaf elongation to GA were observed in KB. The enhanced length of rhizomes by GA_3_ treatment could be associated with the enhanced abundance of proteins and transcription levels regulating energy metabolism (GAPDH, FBA and ATPase), amino acid metabolism (SAM and AH1), protein synthesis (HSP90, HSP70, CPN20, and ELF5A), cell wall development (CDC48C, TUA6, ACT11, and UGDH) and signal transduction (CRT) process, thereby through their integrated actions providing energy products to support cell divisions and subsequent rhizome development in perennial grass species. The biological functions and associated molecular factors of GA-regulated proteins imparting enhanced rhizome elongation deserves further investigation, which will provide new insights to the development of rhizomatous perennial grass species.

## Author Contributions

XM: carry out the experiment and writing; BH: provide research ideas, experimental design, research expenses, and writing.

## Conflict of Interest Statement

The authors declare that the research was conducted in the absence of any commercial or financial relationships that could be construed as a potential conflict of interest.

## Footnotes

1 https://www.arabidopsis.org

2 http://bioinfo.cau.edu.cn/agriGO/analysis.php

3 http://string-db.org/
