# Supplementary material for: Gibberellin-Stimulation of Rhizome Elongation and Differential GA-Responsive Proteomic Changes in Two Grass Species
Source: Front Plant Sci. 2016 Jun 23;7:905. doi: 10.3389/fpls.2016.00905 (PMC4917561; doi:10.3389/fpls.2016.00905)
Supplement: Supplementary file 1 [file Table_1.DOCX]

**TABLE S1**. **Primer sequences used in qRT-PCR.**

| **Gene** | **Forward (5’-3’)** | **Reverse (5’-3’)** | **Size**  **(bp)** | **Accession number** |
| --- | --- | --- | --- | --- |
| *GAPDH*(r344) | GAGGACATCAAGAAGGCTATC | AAGTCAGTGGAGACCAAATC | 92 | GQ480773.1 |
| *CDC48C*(r65) | AGAGGTTGAGAGGCGTATT | TGTTTGGGCGGTTTGTAG | 101 | GT050591.1 |
| *UGDH*(r224) | TAGGGAGACTCCTGCTATTG | CAAGGTCACGCTGGATTT | 107 | GT044309.1 |
| *TUA6*(TF) | CTCTCTGTTGACTATGGAAAGAA | GACAGGACACTGTTGTATGG | 101 | GT042204.1 |
| *CTR*(TF) | AGGACCCAGAAGCTAAGAA | CAGCAACTTCCTTGGGAATA | 108 | GT039428.1 |
| *ACTIN*(TF) | TCTTACCGAGAGAGGTTACTCC | CCAGCTCCTGTTCATAGTCAAG | 107 | AY194227.1^*^ |
| *GAPDH*(k191) | GGTAAGGTTCTTCCTGAGTTG | GCAGCCTTCTCGATTCTAAC | 107 | comp367495_c0 |
| *CDC48C*(k8) | AAGTTGAGCGGCGTATTG | TGTTTGGGCGGTTTGTAG | 99 | comp368959_c0 |
| *UGDH*(KB) | GTCTCACGTCCTCCAATAAG | CATCAACTTCCAGATCCTCTC | 102 | comp351980_c0 |
| *TUA6*(k132) | CCTCAGAGAAGCAGTCAAAG | CGTCGCTCCCTTGATATTG | 90 | comp368530_c0 |
| *CTR*(k101) | ACAGACCGTACACAACATATC | CCCAATCCTTCTGAATCCTATC | 118 | comp366962_c0 |
| *UBQ*(KB) | CCTAGGCGTTGTGCTATATG | CGCCCAAACGTCATACTAA | 91 | comp350900_c1 |
